# Deterministic linkage for improving follow-up time in a Brazilian population-based cancer registry

**DOI:** 10.1038/s41598-023-31303-6

**Published:** 2023-03-24

**Authors:** Talita Fernanda Pereira, Valmir José Aranha, Bernadette Cunha Waldvogel, Allini Mafra da Costa, José Humberto Tavares Guerreiro Fregnani

**Affiliations:** 1 Post Graduate Program of the Education and Research Institute, Barretos Cancer Hospital, Pio XII Foundation, Barretos, São Paulo, 14784-400 Brazil; 2 Based-Population Cancer Registry of Barretos Region, Barretos Cancer Hospital, Pio XII Foundation, Barretos, São Paulo, 14784-400 Brazil; 3 State System of Data Analysis Foundation, São Paulo, 05508-000 Brazil; 4 Department of Precision Health, Luxembourg Institute of Health, 1445 Strassen, Luxembourg; 5 A.C. Camargo Cancer Center, São Paulo, 01525-001 Brazil

**Keywords:** Cancer epidemiology, Cancer epidemiology

## Abstract

Population-based cancer registries (PBCR) are the primary source of cancer incidence and survival statistics. The loss to follow-up of these patients is concerning since it reduces the reliability of any statistical analysis. The linkage techniques have been increasingly used to improve data quality in various information systems. The linkage was performed between the databases of the PBCR-Barretos and the mortality database of the state of São Paulo. To evaluate the improvement in the follow-up time of patients, the comparability of the two databases, pre- and post linkage, was made. Three analyses were performed: a comparative analysis of the absolute number of deaths, a comparative analysis of the follow-up time of patients and the survival analysis. After linkage, there was an increase of 813 deaths. The follow-up time of patients was extended and observed in most types of tumours. The comparability of the survival analyses at both time points also showed a decrease in survival probabilities for all tumour types. Deterministic linkage is effective in updating the vital status of registered patients, improving patient follow-up time, and maintaining good quality data from PBCRs, consequently producing more reliable rates, as seen for the survival analyses.

## Introduction

Population-based cancer registries (PBCRs) are the primary source for cancer incidence and survival statistics and are considered to be the gold standard^1^. These statistics are critical tools for cancer prevention initiatives and are widely regarded as essential for health services and cancer control programs worldwide^2,3^. In addition, survival estimates also contribute to the clinical treatment of patients because, based on the data, doctors can accurately adopt more effective treatments and medications^4^. Therefore, cancer registries should always ensure data quality to avoid inaccurate information about the disease state^2^.

According to the standards recommended by the International Agency for Research on Cancer (IARC), the quality of the PBCR is evaluated according to five dimensions: comparability, validity, timeliness, completeness, and data quality indices for population-based cancer survival. The IARC latest technical report, “Planning and developing population-based cancer registries in low- and middle-income settings," was published in 2014 and described certain quality assessors, such as the quality provided by survival rates, as measured by the follow-up time of cancer cases over time. This feature is mostly dependent on the PBCRs passive follow-up, which involves retrieving reported deaths from vital registry databases^5^. The loss to follow-up (LFU) of these patients is concerning, since it reduces the reliability of statistical analysis and could be a potential bias^6^.

To ensure that these statistics have satisfactory quality, it is necessary to have a complete follow up, from the time of their diagnosis up to their death, or last contact update. Thus, the PBCRs cross-reference their databases with those of civil registries and vital statistics, identifying any deaths and cause of death of patients. However, the linkage process is quite challenging because access to the civil registry databases is often restricted. This restricted access can have numerous causes, the main one being the region's public policies, such as data protection laws, which prevent access to sensitive information about individuals^1^.

Knowing the importance of quality control of the information in the PBCRs, the difficulties these registries face, and the linkage technique as an alternative for improving data quality, the current study aimed to evaluate the effects on the quality of information from the Population-Based Cancer Registry of Barretos, São Paulo, after the deterministic linkage with the mortality database of the State of São Paulo government.

## Material and methods

This observational study included a cohort of 11,346 incident cancer cases in PBCR of Barretos (São Paulo state, Brazil) between 2002 and 2018, with patients ranging in age from 0 to 99 years old and of both sexes.

Through technical cooperation, the mortality database of the State Data Analysis System Foundation (FSEADE) was used for the deterministic type of linkage with the PBCR of incident cancer cases from Barretos. This database has over 3.6 million deaths (excluding fetal deaths) in the State of São Paulo (Brazil). FSEADE used the deterministic technique to detect deaths, to improve follow-up time and to identify new cases (Death Certificate Only—DCO).

To perform the linkage, the database was encrypted and sent to FSEADE through its institutional platform, where the data were sent after registering the researcher's user and password. After the dataset was sent to the platform, only the professionals involved in the linkage had access to download. It was deleted after the affiliated group was allowed.

The name, mother's name, date of birth and Brazilian personal identification number (in Portuguese: Cadastro de Pessoa Física—CPF) were the defining criteria to consider the same individual in both databases (called pairs). Despite all attempts, it was not possible to obtain the mother's name in 91 cases and the date of birth in one case in the PBCR of the Barretos database. The CPF and the individual's name were recorded for all cases in the Barretos RCBP database.

After deterministic linkage, there was a double validation process, which evaluated cases with similar key variables (all cases identified by the system), as well as for doubtful cases (individuals with incomplete records, errors or discrepancies that made the data unreliable or inaccurate in one of the databases, but a high probability of being a match). For these cases, other information was considered, such as: place of birth, residential address, place of death (mainly within the patient's region of origin), and cause of death. After the separate observer evaluation, a third evaluator compared the data and determined whether or not these cases were pairs.

Following the double validation process, the divergent cases between both evaluators were filtered by the system that performed the linkage, where the evaluators together discussed the cases and reached a final decision. The third evaluator was responsible for merging the pairs identified by both the system and the double validation process.

**Figure 1:**
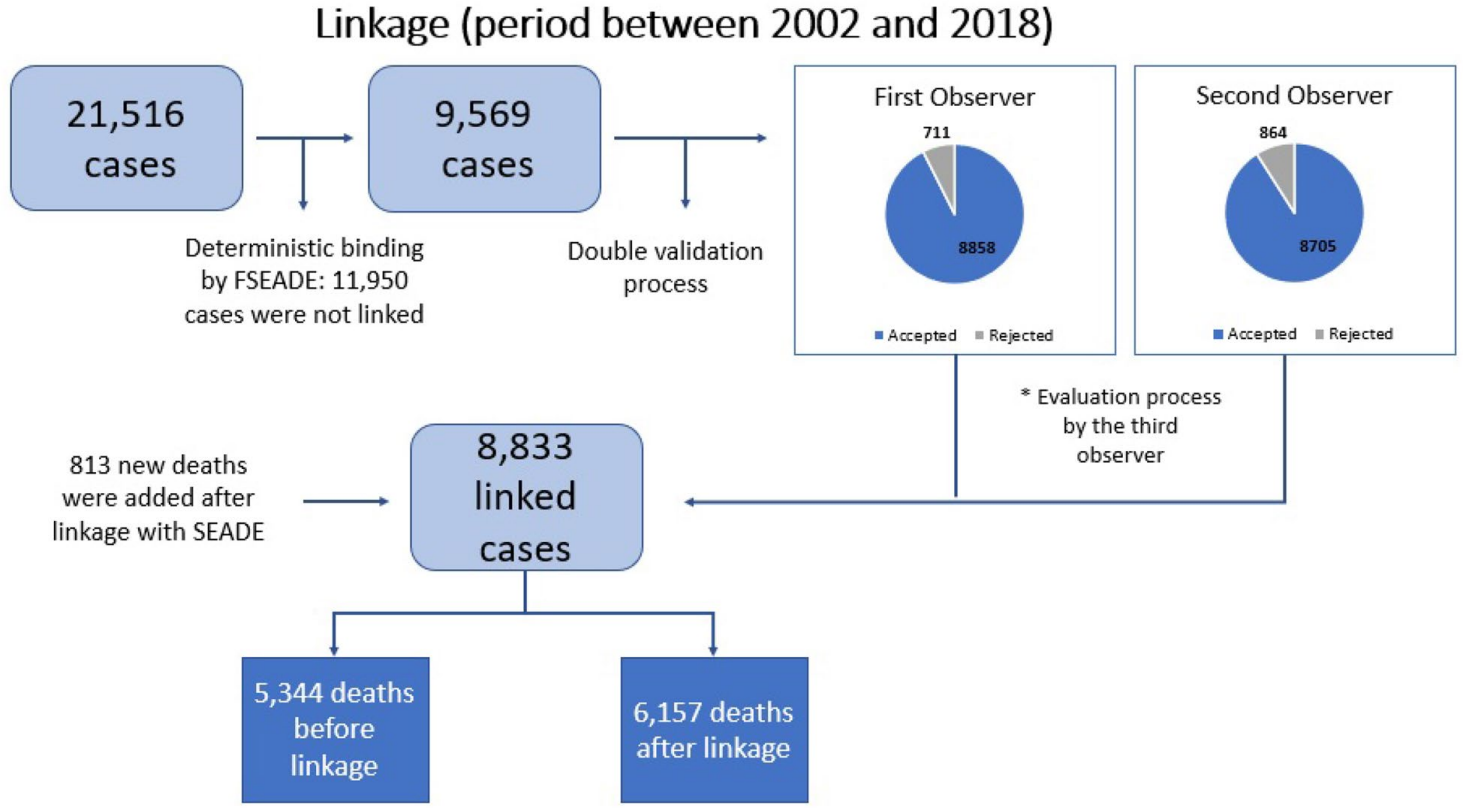
Flowchart with the steps performed in the first deterministic linking and the result of each process.

The linkage process covered the period between 2002 and 2018, containing 21,516 patients. Of these cases, 9566 patients were considered as possible matches, and after the double validation process 8833 patients were considered as true matches (Fig. 1). Additional information can be found in Supplementary Table 1.

The database’s "pre" and "post" deterministic linkage techniques were analysed and compared. After linking, the database was classified by sex, age group, and the 28 leading tumour sites according to the International Statistical Classification of Diseases (ICD-10)^7^: lip (C00), oral cavity (C02-06), pharynx (C01, C09-13), oesophagus (C15), stomach (C16), colon (C18), rectum and rectosigmoid (C19-20), colorectal (C18-20), liver (C22), gallbladder and bile ducts (C23-24), pancreas (C25), larynx (C32), lung and trachea (C33-34), skin melanoma (C43), female breast (C50), cervix uteri (C53), corpus uteri (C54), ovary (C56), prostate (C61), testis (C62), kidney (C64), urinary tract (C65-68), central nervous system (C70-72), thyroid gland (C73), unknown primary site (C80), Hodgkin lymphoma (C81), non-Hodgkin lymphoma (C82-86,96), and leukaemias (C91-95). More information is provided in Supplementary Table 2.

Patients whose only information was a death certificate (Death Certificate Only—DCO) or who were diagnosed at autopsy were removed from the analyses because their survival time was unclear. For the brain, where benign tumours were included (370 cases) (ICD-O Disease Behaviour Code = /0), only primary and invasive tumours were included (ICD-O Disease Behaviour Code = /3). For these cases tumours with benign behaviour were considered for central nervous system tumours, in total in the database, 36 benign tumours were considered for tumour group C70-72, representing 19% of this group.

If the patient was diagnosed with two or more primary tumours in the same anatomical region during the study period (the metachronous primary tumour), only the first tumour identified was included in the analysis. To evaluate the improvement in the quality of patient follow-up, comparability of data before and after deterministic linkage was performed. Four statistical analyses were performed to evaluate the data, including a descriptive analysis of deaths, a descriptive analysis of patient follow-up time, a descriptive analysis of deaths identified after linkage, and a survival analysis.

A descriptive analysis of the absolute number of deaths was performed on both bases (pre- and post linkage). Then, at a second time point, their absolute numbers were compared for each tumour type to determine if there was an increase after the deterministic linkage technique.

Descriptive analysis of patients' follow-up time was performed in two stages. First, a median follow-up time of the patients was performed for each type of tumour, and to analyse whether the differences were significant, the Wilcoxon test was applied. After this analysis, a comparison of the LFU of these patients was performed between the two moments in time. The time of loss to follow-up was categorized into three groups: from 1 to 5 years, 6 to 10 years, and 11 to 15 years after the date of the last contact.

All subjects identified as dead after deterministic linkage underwent a descriptive analysis of their characteristics, including tumour site, clinical stage, age, and the median time (in months) added at the second time point. Tumour sites were classified into main categories using the ICD-10^7^.

Survival analysis was performed using the Kaplan‒Meier nonparametric estimator, and the log-rank test was used, with a significance level of 0.05 established to evaluate the effect of the impact of the deterministic linkage technique between both time points, with “pre” and “post" deterministic linkage techniques. All analyses were carried out using IBM^®^ SPSS^®^ Statistics 20.0.1 software for Windows (IBM Corporation, Route 100, Somers, NY). After the survival analyses, the Wilcoxon test was applied to measure whether there was a significant difference in the two survival curves at a 95% significance level.

This study was conducted after approval by the Research Ethics Committee of the Barretos Cancer Hospital—PIO XII Foundation, under CAAE number: 09247019.3.000.5437. It used retrospective and secondary data from patients in the database of the Population-Based Cancer Registry of Barretos, São Paulo, Brazil. The researchers of this study ensured data confidentiality as well as compliance with the new guidelines of the new Brazilian General Data Protection Regulation^8^.

### Ethics approval and consent to participate

This study was conducted retrospectively utilizing secondary data, and the research participants did not face emotional, intellectual, biological, or physical hazards. However, there are risks of breach of honour, breach of data confidentiality, and access to information. As a result, the researchers of this study ensured the confidentiality of the data collected in alliance with the Research Ethics Committee of the Barretos Cancer Hospital—PIO XII Foundation (CAAE number: 09247019.3.000.5437). The Research Ethics Committee granted a waiver of the Informed Consent Form. It should be noted that the personal data necessary for the implementation of the linkage methodology are not the object of the evaluations of these agreements, but rather the essential characteristics used to link the records of each base. Based on the legal guidelines, the deterministic linkage of data between the databases was performed after the researchers signed the terms of confidentiality and responsibility for the sensitive data. The project also received an addition of guidelines after the new Brazilian General Regulation of Data Protection^8^. All researchers ensured compliance with all sensitive data confidentiality agreements described.

## Results

### Death analysis

**Figure 2:**
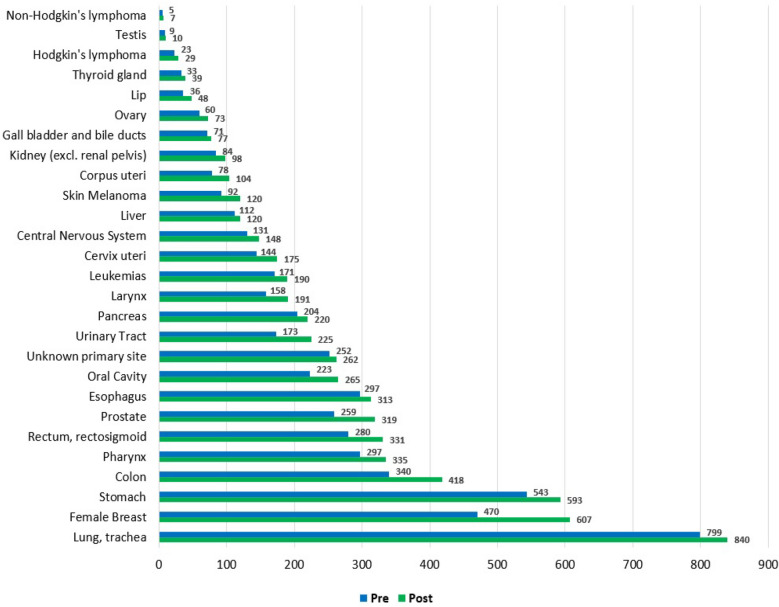
Comparison of the absolute number of deaths from both databases for each tumour site analysed.

We added 813 additional deaths after the deterministic linkage technique (Supplementary Table 2). When the two databases were compared, the tumours with the largest increases in deaths were female breast (137 additional deaths), colon (78 additional deaths) and prostate (60 cases). Conversely, testis cancer (1 additional death), non-Hodgkin’s lymphoma (2 additional deaths), and Hodgkin's lymphoma (6 additional deaths) were the cancer sites with fewer deaths added (Fig. 2).

### Follow-up time

**Figure 3:**
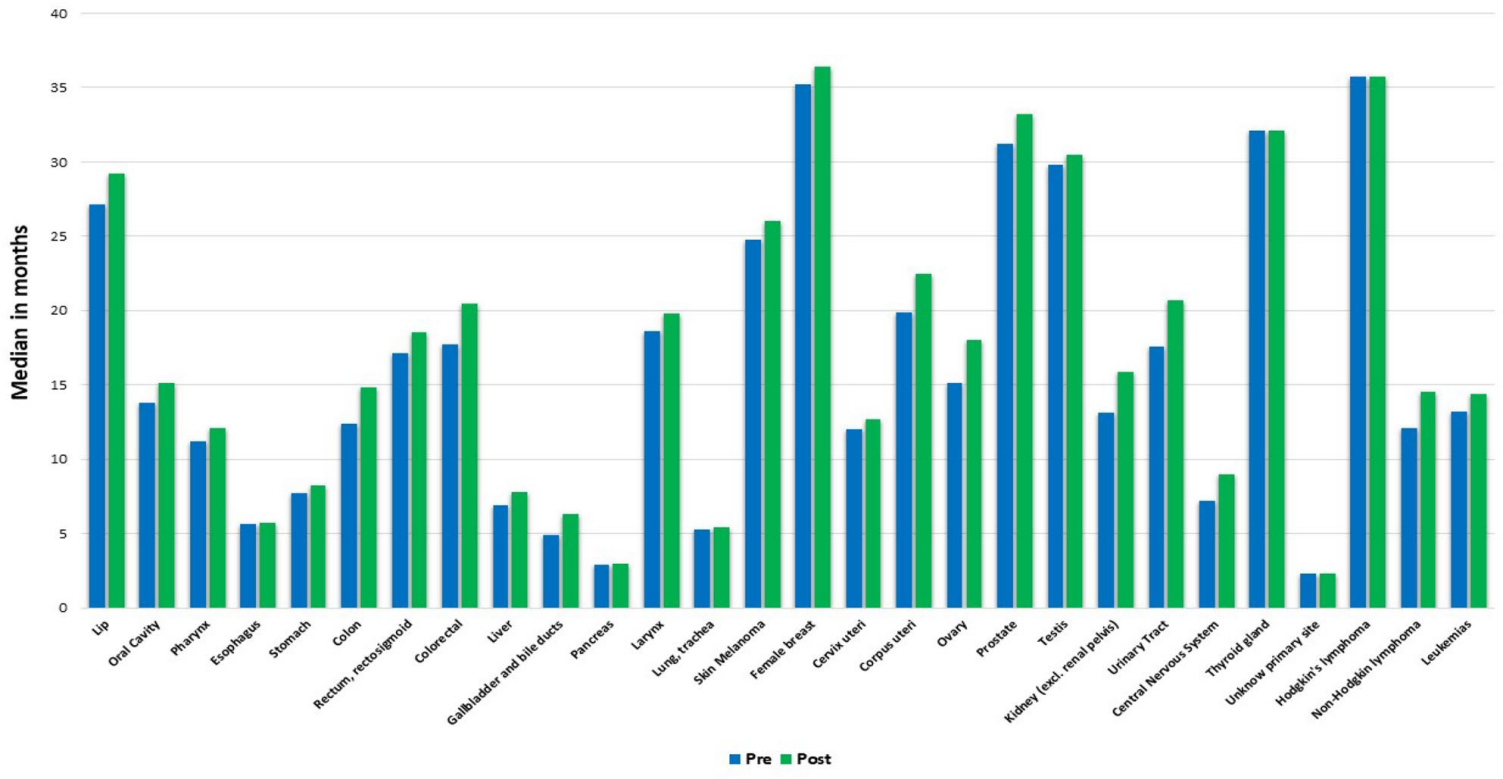
Comparison of the mean follow-up times of individuals for each database categorized by tumour site.

With the additional deaths detected, the dates of the last patient information were updated, increasing the follow-up duration for the patients who had previously been enrolled and correcting certain erroneous death dates for any reason. When the average follow-up time of patients in the two datasets was compared, all tumour types showed significant increases according to their p value, except for non-Hodgkin's lymphoma and testicular cancer (Fig. 3). The results were consistent when using the Wilcoxon test (Supplementary Table 3).

The tumour types that showed the greatest increase in follow-up time were urinary tract cancer (3.1 months added), ovarian cancer (2.9 months added), and kidney cancer (2.9 months added). Conversely, the tumour types that had the least increase in follow-up time were lung and tracheal cancer (0.1 months added), pancreatic cancer (0.1 months added), and stomach cancer (0.5 months added).

### Lost follow-up

**Figure 4:**
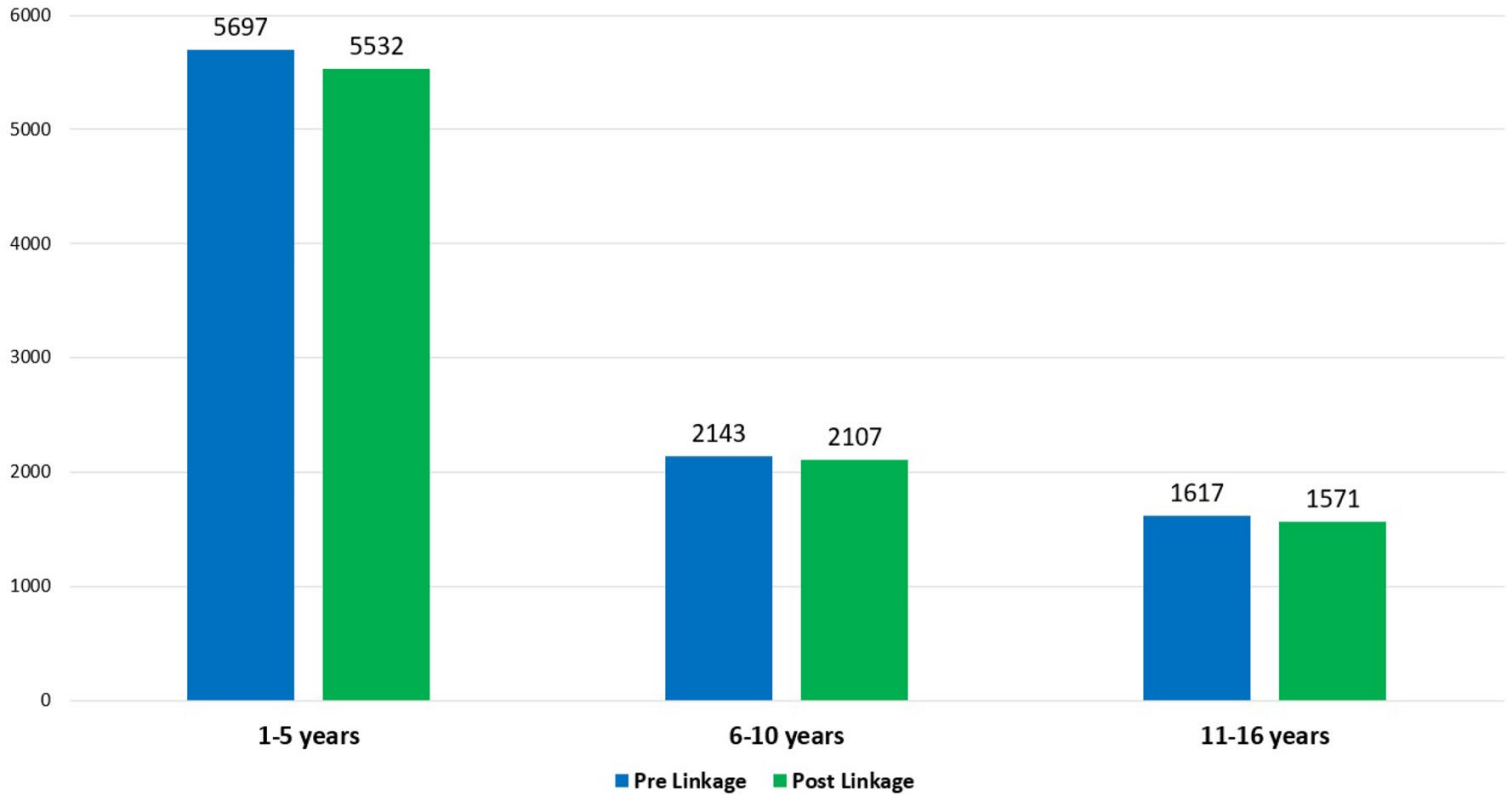
Comparison of the absolute number of lost follow-up times of patients pre- and postlinkage, categorized into the three groups analysed.

The loss of follow-up of patients was analysed considering those who had more than 1 year without contact. At the pre moment, 9457 patients were without contact updates between 1 and 15 years, 60% of them were in the category between 1 and 5 years (5697 cases), 23% were in the category between 6 and 10 years (2143 cases), and 17% were in the category between 11 and 15 years (1617 cases). At the post moment, 9210 patients were without contact updates within the 1- and 15-year periods, 60% of them were in the 1- to 5-year category (5532 cases), 23% were in the 6- to 10-year category (2107 cases), and 17% were in the 11- to 15-year category (1571 cases). Deterministic linkage resulted in the recovery of 247 cases considered as LFU (Fig. 4).

### Overview of the newly identified deaths

Individuals with deaths identified after deterministic linkage were mostly male (53%); among the tumour types analysed, the ones that were most identified as deaths in the linkage were digestive organs (225 cases), female breast cancer (137 cases), and lip, oral cavity, pharynx group (92 cases). The average follow-up time for these patients ranged from 18 to 100 months. For cases identified as deaths after the linkage, the average addition of follow-up time for these patients ranged from the minimum 14 months (Respiratory System and Intratoracic Organs group) to 30 months (Urinary Tract group), as shown in Table 1 below.

**Table 1:** Absolute number of cases as deaths after deterministic linkage stratified by sex, year of diagnosis, and average follow-up time added.

ICD-10	Tumour group	N	Sex		Year of diagnosis			Added follow-up time in months
			Male	Female	2002–2007	2008–2013	2014–2018	
C00-C14	Lip, oral cavity and pharynx	92	72	20	17	23	52	17.0
C15-C26	Digestive organs	225	141	84	56	69	100	22.2
C30-C39	Respiratory system and intrathoracic organs	74	57	17	15	24	35	14.5
C43	Skin melanoma	28	16	12	6	13	9	20.6
C50	Female breast	137	–	137	48	50	39	25.5
C51-C58	Female genital organs	70	–	70	25	21	24	27.4
C60-C63	Male genital organs	61	61	–	28	26	7	27.9
C64-C68	Urinary tract	66	54	12	21	27	18	30.8
C69-C72	Eye, brain and other parts of central nervous system	17	11	6	3	5	9	21.5
C73-C75	Thyroid and other endocrine glands	6	2	4	2	3	1	25.0
C76-C80	Malignant neoplasms of ill-defined, secondary and unspecified sites	10	3	7	4	3	3	16.0
C81-97	Stated or presumed to be primary, of lymphoid, haematopoietic and related tissue	27	14	13	8	12	7	23.8
Total		813	431	382	233	276	304	23.0

### Survival analysis

For the period "pre and post linkage," the population remains the same; only the status patients detected in the linkage with the FSEADE mortality database were updated instead of the alive status, as well as the date of death was updated instead of the date of the last known follow-up. After comparing the one- and five-year survival analyses, a decrease in survival rates was observed for all tumour sites except for testicular cancer. The tumour sites with the greatest decreases in survival rates were female breast, urinary tract, and colorectal cancer (Supplementary Table 4).

**Figure 5:**
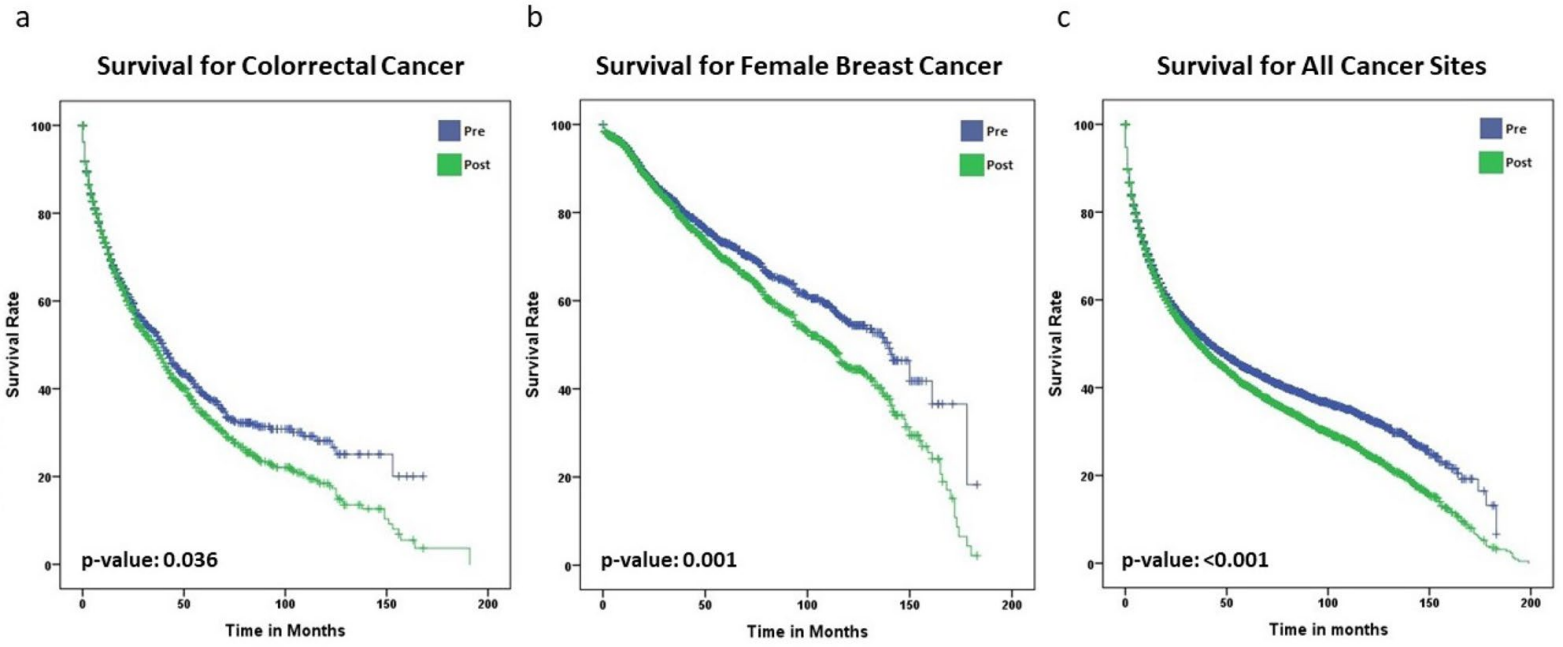
Comparison of the survival curves for (**a**) colorectal cancer, (**b**) female breast cancer, and (**c**) all tumour sites.

When the log rank test was applied, the tumour sites that showed significant decreases in the survival curves were female breast cancer (p-value = 0.042), colorectal cancer (p-value = 0.055), and all cancer sites (p-value < 0.001, excluding non-melanoma skin cancer Fig. 5 shows further survival analysis.

## Discussions

The PBCR of Barretos is located next to the Hospital Cancer Registry of the Barretos Cancer Hospital, which is a reference treatment center and responsible for most of the treatments performed in the Barretos Region. Thus, the PBCR of Barretos is constantly updating the variable of the date of last contact as well as the vital status of individuals since it has easy access to the patient records of the institution. To give continuity to the follow-up of patients' cases, the institution relies on active, and passive follow up. Active follow up is defined as direct contact with the patient or family members, by means of contacts such as telephone. The passive follow up is the one that by indirect means we find updated information, such as notifications of deaths in civil registry offices and medical records of hospital institutions.

However, even with the two types of follow up approaches, many registries remain without updated cases, both because of the great demand of registered cases, where it would be necessary to count on many follow up employees to cover this large number of outdated individuals, and because the means of informed contact are often outdated, making it impossible to perform active follow up. Thus, the linkage with other databases is an alternative to solve these problems in the daily life of PBCR's.

The deterministic linkage between the PBCR of Barretos and the Mortality Database of the State of São Paulo resulted in the match of 41% of cases and added 813 new deaths to the database after linkage. With the imputation of new deaths, the updating of the vital status of patients was performed, demonstrating differences in follow-up time and survival analysis when comparing the two databases, pre- and post linkage.

As a limitation for this study, the linkage with only one database (SEADE mortality database) decreased the chances of pairing cases from the PBCR of Barretos, considering the possibility that the cases registered may go to different states after the diagnosis of cancer, so for subsequent studies, it is suggested that linkage should be performed with different databases and different regions, ensuring a greater population coverage and increasing the chances of a higher percentage of pairing cases.

As a result of the linkage, 41% of the cases in the Barretos PBCR were linked to the mortality database. In other studies, this percentage ranged from 19 to 92%^3,9,10^. Despite the difference in the percentage of linked pairings, the authors met the objectives of the study, always detecting an improvement in the information analysed.

Suzuki et al., aiming to seek confirmation and proof in linkage techniques for information improvement, evaluated the quality and performance of data resulting from probabilistic and deterministic linkages with patient information system data using the three levels of care in the Ribeirão Preto health district in São Paulo, Brazil. The deterministic linkage had a high specificity value when the two relationships were studied and compared. In contrast, the probabilistic relationship was more sensitive and specific, although both effectively boosted data quality^11^.

Even with the high reliability rate of the linkage technique for improving the quality of information, there is great difficulty in performing linkage as a routine procedure by PBCRs, especially in low-income countries. In Brazil, there is difficulty in accessing different databases of vital records, as well as the fact that linkage is a paid, high-cost procedure. The PBCR of Barretos was able to perform this linkage for the first time through the thematic project of this study, which through the financial support of FAPESP, made the linkage with FSEADE possible.

Regarding the improvement in the follow-up time, our results revealed a high rate of significance between the increase in the patients' follow-up time after the linkage. This was due to an increase in deaths found in the database postlinkage and rectification of the last contact and dates of death for these cases. This result has also been reported in the literature; Cherchiglia et al. created a Brazilian database of renal replacement therapy patients using data from the High Complexity Information System (APAC) and linked it to the Outpatient Information System (SAI). The author described the improved follow-up time for renal transplant patients using 34,645,811 cases^12^.

In terms of overall survival rates, which decreased after linkage with FSEADE due to the assignment of deaths in the second database, when the log rank test was applied, there were significant differences in the total case set. Although the difference in survival curves was not statistically significant for the other tumour types, the postlinkage database showed significant improvements over the original database provided by Barretos-PBCR. In addition, correcting the dates of death, updating the vital status of patients (an increase in fatalities), and expanding the follow-up time of patients contributed to improving the quality of the information.

Peres et al. undertook a similar study linking three separate databases and comparing the survival of 122,139 patients in the state of São Paulo before and after linkage. They found an increase of 24,753 database deaths (30 to 38%) after linkage and a decrease in loss to follow-up of 2% (3% to 6%), increasing the cumulative survival of the patients studied (8 to 13%)^9^.

Few studies have been published with the same objective as this one. However, database linkage is increasingly being used to retrieve and improve data in various information systems, with the results being an increase in the mortality and incidence of AIDS patients in Brazil, quantification of the percentage of underreporting of deaths from other diseases, association of risk factors with infant mortality, and improved completeness of patient data such as addresses and dates of birth^9,12,13^. As a result, linking disparate databases is becoming more popular and useful for increasing the quality of information.

Many studies linking multiple information systems have improved the quality of information for a wide range of purposes and conditions. Deterministic linkage between PBCR-Barretos and the FSEADE mortality database, for example, resulted in better information, increased the number of deaths, as shown in the results, and improved the quality of information regarding patients.

## Conclusions

From the results obtained, we can conclude that deterministic linkage effectively improves the follow-up time. However, these processes require financial and human resources, making them inaccessible to many PBCRs. Financial support is needed so that this can be done routinely for all cancer registries. This process was also feasible because of the maintenance of mortality databases in São Paulo. As a suggestion for further studies, the linkage with more than one mortality database from different locations will give a greater probability of identifying more individuals.

## Supplementary Information

Supplementary Tables.

## Data Availability

The datasets used can be made available by deleting information related to the fundamentals from the database. The database can be made available upon request to the corresponding author.

## Abbreviations

PBCR: Population-based Cancer Registries
LFU: Lost follow-up
IARC: International Agency for Research on Cancer
FSEADE: Foundation State System of Data Analysis
DCO: Deaths certificate only
CPF: Brazilian personal identification (in Portuguese: Cadastro de Pessoa Física)
ICD-10: International Statistical Classification of Diseases
APAC: High-complexity information system
SAI: Outpatient information system
SINAN: Notifiable diseases information system
SIM: Mortality information systems
