# Supplementary material for: Deterministic linkage for improving follow-up time in a Brazilian population-based cancer registry
Source: Sci Rep. 2023 Mar 24;13:4816. doi: 10.1038/s41598-023-31303-6 (PMC10039007; doi:10.1038/s41598-023-31303-6)
Supplement: Supplementary file 1 — Supplementary Tables. [file 41598_2023_31303_MOESM1_ESM.docx]

**Supplementary Table 1 –** Processes and results of the two linkages performed between of the PBCR of Barretos database and the FSEADE mortality database.

| **Year of Diagnosis** | **Possible Pairs** | **First Observer** | **Second Observer** | **Difference** | **Linked Cases** |
| --- | --- | --- | --- | --- | --- |
| **2002** | 304 | 279 | 269 | 10 | 279 |
| **2003** | 327 | 290 | 278 | 12 | 290 |
| **2004** | 449 | 404 | 392 | 12 | 402 |
| **2005** | 431 | 392 | 378 | 14 | 389 |
| **2006** | 429 | 412 | 401 | 11 | 412 |
| **2007** | 504 | 468 | 453 | 15 | 466 |
| **2008** | 507 | 488 | 468 | 20 | 486 |
| **2009** | 551 | 513 | 486 | 27 | 513 |
| **2010** | 588 | 554 | 547 | 7 | 548 |
| **2011** | 612 | 572 | 568 | 4 | 568 |
| **2012** | 604 | 577 | 575 | 2 | 577 |
| **2013** | 631 | 591 | 590 | 1 | 591 |
| **2014** | 686 | 634 | 626 | 8 | 633 |
| **2015** | 729 | 678 | 674 | 4 | 678 |
| **2016** | 742 | 684 | 681 | 3 | 683 |
| **2017** | 706 | 632 | 631 | 1 | 633 |
| **2018** | 766 | 687 | 685 | 2 | 685 |
| **Total** | **9,566** | **8,855** | **8,702** | **153** | **8,833** |

**Supplementary Table 2 –** Absolute numbers by cancer site in the "pre" and "post" deterministic linkage databases, as well as the number of deaths in the two databases.

| **ICD-10** | **Cancer site** | **Number of cases** | **Deaths "pre"** | **Deaths “post"** | **Difference**  **N (%)** |
| --- | --- | --- | --- | --- | --- |
| **C00** | Lip | 169 | 36 | 48 | 12 (33.3) |
| **C02-06** | Oral Cavity | 400 | 223 | 265 | 42 (18.8) |
| **C01, C09-13** | Pharynx | 425 | 297 | 335 | 38 (12.7) |
| **C15** | Esophagus | 335 | 297 | 313 | 16 (5.3) |
| **C16** | Stomach | 726 | 543 | 593 | 50 (9.2) |
| **C18** | Colon | 767 | 340 | 418 | 78 (22.9) |
| **C19-20** | Rectum, rectosigmoid | 586 | 280 | 331 | 51 (18.2) |
| **C22** | Liver | 137 | 112 | 120 | 8 (7.1) |
| **C23-24** | Gall bladder and bile ducts | 95 | 71 | 77 | 6 (8.4) |
| **C25** | Pancreas | 233 | 204 | 220 | 16 (7.8) |
| **C32** | Larynx | 281 | 158 | 191 | 33 (20.8) |
| **C33-34** | Lung, trachea | 927 | 799 | 840 | 41 (5.1) |
| **C43** | Skin Melanoma | 322 | 92 | 120 | 28 (30.4) |
| **C50** | Female Breast | 2,040 | 470 | 607 | 137 (29.1) |
| **C53** | Cervix uteri | 526 | 144 | 175 | 31 (21.5) |
| **C54** | Corpus uteri | 249 | 78 | 104 | 26 (33.3) |
| **C56** | Ovary | 132 | 60 | 73 | 13 (21.7) |
| **C61** | Prostate | 893 | 259 | 319 | 60 (23.1) |
| **C62** | Testis | 83 | 9 | 10 | 1 (11.1) |
| **C64** | Kidney (excl. renal pelvis) | 240 | 84 | 98 | 14 (16.6) |
| **C65-68** | Urinary Tract | 417 | 173 | 225 | 52 (30.0) |
| **C70-72** | Central Nervous System | 185 | 131 | 148 | 17 (12.9) |
| **C73** | Thyroid gland | 451 | 33 | 39 | 6 (18.1) |
| **C80** | Unknown primary site | 286 | 252 | 262 | 10 (3.9) |
| **C81** | Hodgkin's lymphoma | 112 | 23 | 29 | 6 (26) |
| **C82-86, C96** | Non-Hodgkin's lymphoma | 16 | 5 | 7 | 2 (40) |
| **C91-95** | Leukemias | 313 | 171 | 190 | 19 (11.1) |
| **Total** | | 11,346 | 5,344 | 6,157 | 813 (15.2) |

**Supplementary Table 3 –** Median, maximum, and minimum of follow-up (in months) of patients in the "pre" and "post" deterministic linkage databases by cancer site.

| **ICD-10** | **Cancer site** | **Moment** | **Median (months)** | **Difference** | **Minimum (months)** | **Maximum (months)** | **p-value*** |
| --- | --- | --- | --- | --- | --- | --- | --- |
| **C00** | Lip | Pre | 27.1 | +2.1 | 0.03 | 166.7 | 0.002 |
|  |  | Post | 29.2 |  | 0.03 | 166.7 |  |
| **C02-06** | Oral cavity | Pre | 13.8 | +1.3 | 0.13 | 183.5 | < 0.001 |
|  |  | Post | 15.1 |  | 0.13 | 194.3 |  |
| **C01, C09-13** | Pharynx | Pre | 11.2 | +0.9 | 0.23 | 140.5 | < 0.001 |
|  |  | Post | 12.1 |  | 0.23 | 155.7 |  |
| **C15** | Esophagus | Pre | 5.6 | +0.1 | 0.03 | 143.6 | < 0.001 |
|  |  | Post | 5.7 |  | 0.03 | 143.6 |  |
| **C16** | Stomach | Pre | 7.7 | +0.5 | 0.00 | 159.7 | < 0.001 |
|  |  | Post | 8.2 |  | 0.00 | 159.7 |  |
| **C18** | Colon | Pre | 12,4 | +2.4 | 0.13 | 163.0 | < 0.001 |
|  |  | Post | 14.8 |  | 0.13 | 191.2 |  |
| **C19-20** | Rectum, rectosigmoid | Pre | 17.1 | +1.4 | 0.13 | 168.6 | < 0.001 |
|  |  | Post | 18.5 |  | 0.13 | 168.6 |  |
| **C18-20** | Colorectal | Pre | 17.7 | +2.8 | 0.13 | 168.6 | < 0.001 |
|  |  | Post | 20.5 |  | 0.13 | 191.2 |  |
| **C22** | Liver | Pre | 6.9 | +0.9 | 0.03 | 123.0 | 0.003 |
|  |  | Post | 7.8 |  | 0.13 | 123.0 |  |
| **C23-24** | Gallbladder and bile ducts | Pre | 4.9 | +1.4 | 0.07 | 123.9 | 0.012 |
|  |  | Post | 6.3 |  | 0.07 | 131.7 |  |
| **C25** | Pancreas | Pre | 2.9 | +0.1 | 0.07 | 102.4 | 0.001 |
|  |  | Post | 3.0 |  | 0.07 | 102.4 |  |
| **C32** | Larynx | Pre | 18.6 | +1.2 | 0.10 | 166.3 | < 0.001 |
|  |  | Post | 19.8 |  | 0.69 | 166.3 |  |
| **C33-34** | Lung, trachea | Pre | 5.3 | +0.1 | 0.03 | 156.8 | < 0.001 |
|  |  | Post | 5.4 |  | 0.03 | 199.7 |  |
| **C43** | Skin melanoma | Pre | 24.8 | +1.2 | 0.00 | 177.6 | < 0.001 |
|  |  | Post | 26.0 |  | 0.00 | 177.6 |  |
| **C50** | Female breast | Pre | 35.2 | +1.2 | 0.16 | 183.4 | < 0.001 |
|  |  | Post | 36.4 |  | 0.16 | 183.4 |  |
| **C53** | Cervix uteri | Pre | 12.0 | +0.7 | 0.00 | 155.2 | < 0.001 |
|  |  | Post | 12.7 |  | 0.00 | 190.3 |  |
| **C54** | Corpus uteri | Pre | 19.9 | +2.6 | 0.43 | 170.3 | < 0.001 |
|  |  | Post | 22.5 |  | 0.43 | 172.2 |  |
| **C56** | Ovary | Pre | 15.1 | +2.9 | 0.00 | 145.6 | 0.001 |
|  |  | Post | 18.0 |  | 0.00 | 145.6 |  |
| **C61** | Prostate | Pre | 31.2 | +2.0 | 0.07 | 174.8 | < 0.001 |
|  |  | Post | 33.2 |  | 0.07 | 175.8 |  |
| **C62** | Testis | Pre | 29.8 | +0.7 | 0.03 | 131.8 | 0.317 |
|  |  | Post | 30.5 |  | 0.03 | 178.4 |  |
| **C64** | Kidney (excl. Renal pelvis) | Pre | 13.1 | +2.8 | 0.03 | 150.8 | < 0.001 |
|  |  | Post | 15.9 |  | 0.03 | 192.5 |  |
| **C65-68** | Urinary tract | Pre | 17.6 | +3.1 | 0.03 | 181.4 | < 0.001 |
|  |  | Post | 20.7 |  | 0.03 | 191.8 |  |
| **C70-72** | Central nervous system | Pre | 7.2 | +1.8 | 0.03 | 150.2 | < 0.001 |
|  |  | Post | 9.0 |  | 0.03 | 150.2 |  |
| **C73** | Thyroid gland | Pre | 32.1 | 0 | 0.07 | 156.4 | 0.018 |
|  |  | Post | 32.1 |  | 0.07 | 178.3 |  |
| **C80** | Unknown primary site | Pre | 2.3 | 0 | 0.03 | 136.8 | 0.004 |
|  |  | Post | 2.3 |  | 0.03 | 156.6 |  |
| **C81** | Hodgkin's lymphoma | Pre | 35.7 | 0 | 0.26 | 153.4 | 0.028 |
|  |  | Post | 35.7 |  | 0.26 | 169.4 |  |
| **C82-86, C96** | Non-Hodgkin lymphoma | Pre | 12.1 | +2.4 | 0.07 | 96.7 | 0.180 |
|  |  | Post | 14.5 |  | 0.07 | 96.7 |  |
| **C91-95** | Leukemia | Pre | 13,2 | +1,2 | 0.03 | 153.5 | < 0.001 |
|  |  | Post | 14,4 |  | 0.03 | 154.9 |  |
|  | **Total** | Pre | 14.5 | +1.2 | 0.03 | 183.5 | < 0.001 |
|  |  | Post | 15.7 |  | 0.03 | 199.7 |  |

**SD: Standard Deviation * Wilcoxon test. Supplementary Table 4 –** Overall survival for the "pre" and "post" databases by cancer site.

| **ICD - 10** | **Cancer site** | **Survival** | | | | | | **p-value*** |
| --- | --- | --- | --- | --- | --- | --- | --- | --- |
|  |  | **1-year Pre** | **1-year Post** | **Difference** | **5-year Pre** | **5-year Post** | **Difference** |  |
| **C00** | Lip | 98.0 | 95.7 | - 2.3 | 66.4 | 62.8 | - 3.6 | 0.325 |
| **C02-06** | Oral cavity | 68.2 | 66.1 | - 2,1 | 36.3 | 31.5 | - 4.8 | 0.146 |
| **C01. C09-13** | Pharynx | 57.1 | 56.4 | - 0.7 | 17.5 | 14.1 | - 3.4 | 0.381 |
| **C15** | Esophagus | 25.4 | 24.7 | - 0.7 | 5.0 | 3.6 | - 1.4 | 0.676 |
| **C16** | Stomach | 43.2 | 42.7 | - 0,5 | 13.9 | 12.2 | - 1.7 | 0.449 |
| **C18** | Colon | 72.9 | 72.9 | 0 | 39.7 | 35.7 | - 4.0 | 0.149 |
| **C19-20** | Rectum. Rectosigmoid | 73.9 | 73.6 | - 0.3 | 38.4 | 32.9 | - 5.5 | 0.124 |
| **C18-20** | Colorectal | 72.4 | 72.4 | 0 | 38.3 | 33.9 | - 4.4 | 0.036 |
| **C22** | Liver | 36.6 | 35,1 | - 1.5 | 8.9 | 7.5 | - 1.4 | 0.866 |
| **C23-24** | Gall bladder and bile ducts | 40.1 | 39.4 | - 0.7 | 13.4 | 13.3 | - 0.1 | 0.967 |
| **C25** | Pancreas | 17.0 | 16.9 | - 0.1 | 4.9 | 3.5 | - 1.4 | 0.756 |
| **C32** | Larynx | 75.9 | 75.7 | - 0.2 | 38.7 | 31.9 | - 6.8 | 0.186 |
| **C33-34** | Lung. Trachea | 31.7 | 29.9 | - 0.4 | 7.1 | 5.9 | - 1.2 | 0.694 |
| **C43** | Skin melanoma | 90.0 | 89.5 | - 0.1 | 63.8 | 59.0 | - 4.8 | 0.123 |
| **C50** | Female breast | 95.1 | 94.9 | - 0.2 | 73.2 | 69.5 | - 3.7 | 0.001 |
| **C53** | Uterine corpus | 83.8 | 83.3 | - 0.5 | 54.7 | 49.9 | - 4.8 | 0.224 |
| **C54** | Uterine body | 86.6 | 85.7 | - 0.9 | 53.4 | 52.7 | - 0.7 | 0.151 |
| **C56** | Ovary | 73.2 | 71.2 | - 2.0 | 44.5 | 40.4 | - 4.1 | 0.472 |
| **C61** | Prostate | 93.8 | 93.4 | - 0.4 | 67.8 | 65.4 | - 2.4 | 0.076 |
| **C62** | Testis | 96.6 | 96.6 | 0 | 89.8 | 89.8 | 0 | 1.000 |
| **C64** | Kidney (excl. Renal pelvis) | 78.3 | 77.9 | - 0.4 | 51.3 | 47.7 | - 3.6 | 0.626 |
| **C65-68** | Urinary tract | 76.0 | 75.1 | - 0.9 | 51.8 | 46.1 | - 5.7 | 0.074 |
| **C70-72** | Central nervous system | 45.8 | 45.1 | - 0.7 | 20.6 | 18.1 | - 2.5 | 0.626 |
| **C73** | Thyroid gland | 97.4 | 97.1 | - 0.3 | 90.1 | 89.5 | - 0.6 | 0.582 |
| **C80** | Unknown primary site | 24.0 | 23.4 | - 0.6 | 9.5 | 8.1 | - 1.4 | 0.746 |
| **C81** | Hodgkin's lymphoma | 87.4 | 87.4 | 0 | 78.0 | 76.9 | - 1.1 | 0.529 |
| **C82-86. C96** | Non-Hodgkin lymphoma | 72.9 | 72.2 | - 0.7 | 48.6 | 47.6 | - 1.0 | 0.751 |
| **C91-95** | Leukemias | 63.2 | 62.9 | - 0.4 | 40.8 | 38.4 | - 2,4 | 0.584 |
| **Total** | | 70.4 | 69.9 | - 0.5 | 44.6 | 40.7 | - 3.9 | <0.001 |

*** Log-Rank test.**
